# A mendelian randomization study on causal effects of 25(OH)vitamin D levels on attention deficit/hyperactivity disorder

**DOI:** 10.1007/s00394-020-02439-2

**Published:** 2020-11-27

**Authors:** Lars Libuda, Roaa Naaresh, Christine Ludwig, Björn-Hergen Laabs, Jochen Antel, Manuel Föcker, Johannes Hebebrand, Anke Hinney, Triinu Peters

**Affiliations:** 1 Department of Child and Adolescent Psychiatry, Psychosomatics and Psychotherapy, University Hospital Essen, University of Duisburg-Essen, Virchowstr. 174, 45147 Essen, Germany; 2 Research Institute for the Prevention of Allergies and Respiratory Diseases in Childhood, Department of Pediatrics, Marien-Hospital Wesel, 46483 Wesel, Germany; 3 Institut für Medizinische Biometrie und Statistik, Universität zu Lübeck, Universitätsklinikum Schleswig-Holstein, 23562 Lübeck, Germany; 4 Department of Child and Adolescent Psychiatry, University of Münster, 48149 Münster, Germany

**Keywords:** Vitamin D, 25(OH)vitamin D, ADHD, Attention, Mendelian randomization, Prevention

## Abstract

**Background:**

While observational studies revealed an inverse association between serum 25(OH)vitamin D (25(OH)D) and the risk of attention deficit/hyperactivity disorder (ADHD), the causality of this relationship remains unclear.

**Methods:**

We conducted a bidirectional two-sample Mendelian Randomization (MR) study to examine whether 25(OH)D has an effect on the risk to develop ADHD or vice versa. Information on single nucleotide polymorphisms (SNP) associated with serum 25(OH)D was obtained from a genome-wide association study (GWAS) considering phenotype data from 79,366 individuals of European ancestry. Data on risk for ADHD were derived from a GWAS analysis with 20,183 individuals diagnosed with ADHD and 35,191 controls. For our analysis, we considered effect sizes based on the European participants (19,099 cases and 34,194 controls).

**Results:**

Single SNP analyses showed a causal effect of vitamin D on ADHD risk for only one SNP (rs12785878, *p* = 0.024). The overall MR estimates did not reveal a causal effect of 25(OH)D on risk for ADHD. In the reverse analysis, neither any single nor the multi-SNP MR analyses showed a causal effect of ADHD on 25(OH)D.

**Conclusion:**

Results from this two-sample MR study did not confirm a causal effect of 25(OH)D on ADHD or vice versa. Accordingly, our study does not provide evidence that improving 25(OH)D via supplementation could reduce the risk of developing ADHD.

**Electronic supplementary material:**

The online version of this article (10.1007/s00394-020-02439-2) contains supplementary material, which is available to authorized users.

## Introduction

Considering its impact on calcium and phosphate metabolism, vitamin D had initially been discussed in particular in the context of bone health [1]. While European scientific societies already recommend vitamin D supplementation during infancy [2], recent findings from observational studies on potential negative non-skeletal effects of vitamin D deficiency caused a debate on the meaningfulness of population-wide recommendations for vitamin D supplementation in childhood and adolescence [3]. Further, the role of vitamin D for mental health might be important since it is considered as one example of a nutrient supplement that could be beneficial in the management of mental disorders [4].

In a systematic literature search regarding childhood and adolescence, we recently identified a large number of (predominantly observational) studies dealing with the relationship between vitamin D and mental health [5]. Besides autism spectrum disorder, most of these studies focused on attention deficit/hyperactivity disorder (ADHD). Results from a recent genetic look-up analysis, based on data from large-scaled genome-wide associations studies (GWAS), revealed a genetic variant which is concomitantly associated with both vitamin D insufficiency and ADHD [6]. This genetic overlap might indicate a direct association.

In general, vitamin D effects on mental health seem plausible considering the fact that both vitamin D receptors and metabolizing enzymes are located in the brain [7]. With regard to ADHD, several pathways are currently discussed such as anti-inflammatory and anti-oxidative actions or an influence on neurotrophic factors and neurotransmitter metabolism [8]. A recent randomized controlled trial (RCT) in children with ADHD showed that supplementation of 2000 IU vitamin D per day for 12 weeks resulted in significantly increased serum dopamine levels in the intervention group compared to the placebo group while serum brain-derived neurotrophic factor and serotonin levels did not change [9].

A meta-analysis recently summarized results from four RCTs with vitamin D supplementation in ADHD patients and revealed a small, but significant improvement of ADHD symptoms [10]. However, all considered RCTs were conducted in children with an already manifest ADHD, enabling conclusions only on therapeutic, but not on preventive effects of vitamin D. However, RCTs to detect preventive effects would require very large sample sizes to provide sufficient statistical power as well as long-term follow-up periods [11] to cover a critical time frame of disorder pathogenesis.

In contrast, Mendelian randomization (MR) studies based on summary data from GWAS are a time-effective approach using genetic variants as instrumental variables (IVs) to examine the causal effect of blood concentrations of a specific nutrient (e.g. vitamin D) on a particular disorder (e.g. ADHD) [12]. The concept of MR studies implies a natural “quasi randomization”, since the individual composition of alleles and, thus, of IVs are determined randomly at conception, resulting in a reduced risk of confounding [13]. Bias from reverse causation, another limitation of observational studies, is also precluded in MR studies, as the individual genotype is determined at conception, and cannot be modified by the outcome of interest [13].

Genetic factors play an important role for levels of circulating vitamin D [14] with heritability estimates based on twin studies varying from 39 to 70% for Caucasians and 86% in a community-based study of adolescents (mean age 16 years) [14, 15]. Accordingly, MR studies seem to be particularly suitable to evaluate health effects of serum 25(OH) vitamin D levels (25(OH)D). Regarding the role of vitamin D for mental disorders, MR studies recently did not confirm the discussed effects on depression [16, 17]. More recently, a phenome-wide MR study using data from UK biobank revealed no evidence of causal effects of 25(OH)D on a large number of outcomes including depression, nonvertebral fracture, and all-cause mortality [18]. Since MR studies which focus on effects on ADHD have not been conducted, the aim of this two-sample MR study was to examine the effects of 25(OH)D on ADHD using summary-level data of recent large-scaled GWAS on 25(OH)D levels and ADHD. A reverse MR analysis on causal effects of ADHD on 25(OH)D was conducted to examine whether associations found in observational studies might represent reverse causation.

## Methods

### Data sources for MR analyses and selection of the genetic instruments

We conducted a two-sample MR analysis with 25(OH)D as exposure and ADHD as outcome variable and a reverse analysis with ADHD as exposure and level of 25(OH)D as outcome. Information on single nucleotide polymorphisms (SNPs) associated with serum 25(OH)D were obtained from the latest GWAS analysis at the time of our data analysis considering phenotype data from 79,366 individuals of European ancestry including 31 studies from epidemiological cohorts from Europe, Canada, and USA [19]. We used independent SNPs of all six loci with genome-wide significance for 25(OH)D as the genetic instrument. The effect estimates of these genetic variants on the exposure were derived from the publicly available summary statistics of the GWAS meta-analysis. The six 25(OH)D-associated SNPs explained 2.84% of the variance of serum 25(OH)D levels [19].

Data on risk for ADHD were derived from a GWAS analysis with 20,183 individuals diagnosed with ADHD and 35,191 controls from 12 cohorts [20]. Twelve loci with genome-wide significance were identified. The SNP-heritability was 21.6%. For our analysis, we considered effect estimates from European participants (19,099 cases and 34,194 controls). Two-sample MR assumes independent samples. The screening of the study groups in the GWAS for 25(OH)D and ADHD showed no sample overlap.

In case of unavailability of SNPs identified in the 25(OH)D GWAS in the ADHD GWAS or vice versa, we used proxy-SNPs as recommended [21]. For the search of proxies, SNPs with minimum linkage disequilibrium (LD) r^2^ ≥ 0.40, on the basis of GRCh37.p13, Ensembl version 87, 1000 genomes: phase 3 version 5 for European ancestry, were exported applying the in silico tool SNiPA [22] (http://www.snipa.org. Accessed on 19 August 2019). Post-hoc selection criteria for proxy-SNPs were defined: 1st highest r^2^, 2nd smallest distance to the lead SNP. In the sensitivity analysis for reverse MR, we selected the proxy SNPs with the following selection criteria: 1st highest r^2^, 2nd smallest distance to the lead SNP, 3rd no palindromic alleles. Because of the potential problems with the strand [21], we excluded SNPs, which were both palindromic and ambiguous.

During the review process of this paper, two additional GWAS on the impact of genetic variation on 25(OH)D concentrations revealed substantial larger numbers of genome-wide significant loci. Revez and colleagues used data from 417,580 European participants from UK Biobank and observed 143 independent loci [23]. Manousaki et al. combined data from 401,460 UK Biobank participants with data from 42,274 Europeans from a previous GWAS and observed 138 conditional independent SNPs in 69 independent loci [24]. These increased numbers of genome-wide significant SNPs could contribute to a stronger genetic instrument. However, Manousaki et al. concluded that several SNPs identified in their GWAS were mapped to genes not directly involved in 25(OH)D metabolism and could, thus, increase the risk of pleiotropic effects [24]. In contrast, four out of the six SNPs identified in the SUNLIGHT GWAS are related to genes involved in established pathways of vitamin D metabolism. Accordingly, we kept the results based on the SUNLIGHT GWAS as main analysis and provided findings based on genetic instruments derived from the two latter GWAS as additional sensitivity MR analyses.

For these two sensitivity analyses, we defined an alternative genetic instrument based on results from the respective GWAS on 25(OH)D and conducted separate MR analyses. Only those SNPs from the respective GWAS on 25(OH)D were considered which were both available in the ADHD GWAS and not ambiguous or palindromic. This requirement was met by 100 from 143 genome-wide significant SNPs identified by Revez et al. and 61 from 138 SNPs identified by Manousaki and colleagues.

### Testing MR assumptions and statistical analysis

As recently suggested [25] we used several statistical methods for the calculation of an overall causal effect estimate of 25(OH)D on ADHD (and vice versa): Inverse Variance Weighted (IVW) and MR-Egger as main analyses [26] and Weighted Median [27] as well as mode-based estimators (MBE) [25] as sensitivity analyses. These methods make different assumptions and can, thus, be used to assess the robustness of MR results against violations of the instrumental variable assumptions. IVW assumes that all ratio estimates provide independent evidence on the causal effect and there is no pleiotropic effect. The IVW accordingly assumes that all genetic variants are valid instrumental variables. There is no intercept term in the regression model [26]. In MR-Egger the intercept term is estimated as part of the analyses and can be interpreted as the average pleiotropic effect of a genetic variant included in the analyses [26]. Weighted Median estimator is consistent even when up to 50% of the information comes from invalid instrumental variables [27]. The MBE is consistent even if the majority of instruments are invalid. Its power to detect a causal effect is smaller compared with the IVW and weighted median methods, but larger than that of MR-Egger regression [25].

To conduct a MR study, three core assumptions must be fulfilled [12]: (1) The genetic instrument must have a true association with the exposure. To fulfill this assumption we used independent genome-wide significantly associated SNPs for 25(OH)D (*p* < 5 × 10^–8^). For reverse analysis with ADHD as exposure, we used SNPs which were genome-wide significantly associated with ADHD (*p* < 5 × 10^–8^) with the risk of ADHD as the genetic instrument. (2) There is no association between the genetic variants and confounders of the relationship between risk factor and outcome. (3) Conditioning on the risk factor and possible confounders, there is no direct association between the genetic variants and outcome [12]. Thus, the effect of the genetic instrument on the outcome must be mediated exclusively by the exposure and there must be no direct effects [28].

From these assumptions, only the first assumption can be directly tested [28]. For this purpose, we conducted an F-test to test the weakness of the instrument. If F-statistic is less than ten, the instrument variable is considered weak [29]. For examination of assumptions 2 and 3, multiple approaches were applied. We examined horizontal pleiotropy by estimating the intercept of Egger's regression. If Eggers intercept is not significantly different from zero, it can be assumed that there is no horizontal pleiotropy [26]. Additionally, we used MR-PRESSO to identify horizontal pleiotropic outliers and to calculate an overall outlier-corrected causal estimate [30]. Genetic polymorphisms are sometimes associated with multiple aspects or dimensions of a single trait [28]. To test such heterogeneity of the instrument variable, we used Cochran’s Q-statistic with the null hypothesis is that the MR-Egger regression model describes the associations with the outcome with no excess heterogeneity [31]. This test examines whether causal estimates of genetic variants (SNPs) are comparative [32]. To investigate the relationship between study accuracy and effect size, we created a funnel plot [28]. Asymmetry in the funnel plot indicates that assumptions for MR are not met [33]. To examine whether an individual data point (SNP) has a large influence on the regression coefficients, we conducted a leave-one-out approach. For this analysis, we conducted the IVW regression by leaving each genetic variant out in turn [26].

Forest and scatter plots were used to visualize combined results of single and multi-SNP analyses. The scatter plots show the single SNP effects on the exposure against the single SNP effects on the outcome with corresponding standard deviations and estimated regression lines of the multi-SNP analyses. We performed a power analysis to estimate whether our analysis, given sample size, proportion of cases in the study, and the proportion of variance explained, provides sufficient statistical power to detect a true causal effect [34].

All tests were performed using the software “R “, version 3.5.2, and R-packages for performing 2-sample MR (https://github.com/MRCIEU/TwoSampleMR) and MR-PRESSO (https://github.com/rondolab/MR-PRESSO).

## Results

### MR assessing 25(OH)D effects on ADHD

We performed a MR analysis to investigate the causal effect of 25(OH)D levels on risk of ADHD. The data on the six SNPs associated with the genome-wide significance of *p* < 5 × 10^–8^ with 25(OH)D are presented in Table 1. For one of these SNPs, a proxy SNP (rs209955) was considered due to missing information in the ADHD GWAS (rs17216707). One ambiguous and palindromic SNP (rs8018720) was not included in the analysis resulting in five SNPs used for the definition of the IV (Table 1).

**Table 1:** Genome-wide significant single nucleotide polymorphisms (SNPs) for natural log-transformed 25(OH)D levels and their association with ADHD

SNP		25(OH)D						ADHD					Palindromic + ambiguous
		A1	A2	Eaf (A1)	Beta	SE	*P* value	A1	A2	OR	SE	*P* value	
rs10741657	CYP2R1	A	G	0.40	0.031	0.0022	2.05E–46	A	G	1.0048	0.0137	0.728	False
rs10745742	AMDHD1	T	C	0.40	0.017	0.0022	1.88E–14	T	C	1.0141	0.0138	0.311	False
rs12785878	NADSYN1-DHCR7	T	G	0.75	0.036	0.0022	3.80E–62	T	G	1.0313	0.0146	0.035	False
*rs17216707*^*a*^	*CYP24A1*	*T*	*C*	*0.77*	*0.026*	*0.0027*	*8.14E–23*						
^a^rs209955		T	C	0.31^b^	− 0.019	0.0024	2.49E−16	T	C	1.003	0.0156	0.848	False
rs3755967	GC	T	C	0.28	− 0.089	0.0023	4.74E–343	T	C	1.0235	0.015	0.122	False
rs8018720	SEC23A	C	G	0.82	− 0.017	0.0029	4.72E–09	C	G	0.9958	0.0185	0.819	True

*Eaf* effect allele frequency, *OR* odds ratio, *SE* standard error, *palindromic* + *ambiguous true* this SNP is palindromic and ambiguous and will be excluded

^a^rs209955 is the proxy for rs17216707: distance = − 9491, *D*′ = 0.7908, *r*^2^ = 0.4115; A1: Allele 1; A2: Allele 2

^b^Eaf (European) from https://ldlink.nci.nih.gov/?tab=ldhap

**Fig. 1:**
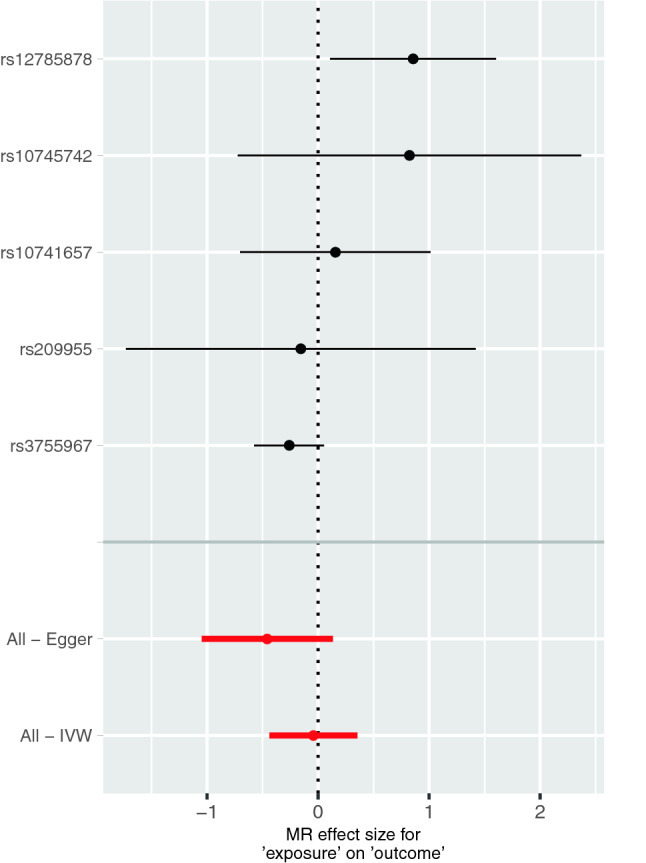
Results of the single and multi-SNP analyses for the SNP effect of natural-log transformed 25(OH)D levels on ADHD. The black lines visualize the results of single SNP analyses; the red lines visualize the results of the multi-SNP analysis

**Fig. 2:**
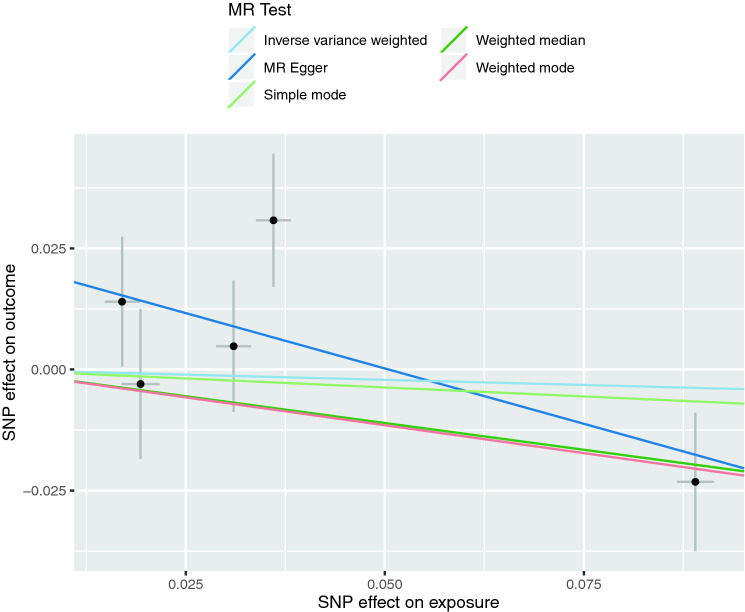
Scatterplots of genetic associations with natural-log transformed 25(OH)D levels against risk for ADHD using different MR methods. The slopes of each line represent the causal association for each method

With *F* = 1558.762, the F-statistic indicates strong instrumental variables (variance explained by the six SNPs (2.84%) was used for the calculation). There was no evidence for pleiotropy (MR-Egger intercept: 0.0231, SE = 0.0139; *p* = 0.1955) or heterogeneity (Cochran's Q (*df* = 3) = 4.594, *p* = 0.204). MR-PRESSO also revealed no evidence for horizontal pleiotropy. Single SNP MR analyses showed that only SNP rs12785878 *(NADSYN1-DHCR7)* was associated with ADHD. The overall estimates calculated by IVW or MR-Egger, did not reveal an overall causal effect of 25(OH)D levels on the risk of ADHD (Table 2, Figs. 1, 2, and S1). Sensitivity analyses using Weighted Median, Simple Mode, and Weighted Mode confirmed the lack of associations (Table 2, Fig. 2). MR-PRESSO also did not show a significant association between 25(OH)D and ADHD (*β* = − 0.043, SD = 0.202, *p* = 0.843). However, the leave-one-out analysis showed that the exclusion of SNP rs3755967 (*GC*: group-specific component gene, GC protein is a major vitamin D-binding protein in plasma [35]) would lead to a significant overall positive effect of 25(OH)D on ADHD, i.e. higher 25(OH)D levels would cause higher ADHD risk (Figure S2). Power analyses revealed that our MR analyses had 80% power to detect an OR of 1.159 for ADHD per 1 standard deviation decrease in natural-log transformed 25(OH)D levels (Figure S3).

**Table 2:** Results of single SNP MR analyses and the overall causal effect of natural-log transformed 25(OH)D levels on the risk of ADHD calculated using different statistical methods

SNP	Beta	SE	*P* value
rs10741657	0.155	0.438	0.724
rs10745742	0.824	0.789	0.297
rs12785878	0.856	0.382	0.024
rs209955	− 0.155	0.803	0.847
rs3755967	− 0.261	0.161	0.105
Inverse variance weighted	− 0.043	0.202	0.833
MR Egger	− 0.457	0.301	0.227
Weighted median	− 0.221	0.158	0.162
Simple mode	− 0.074	0.466	0.881
Weighted mode	− 0.230	0.161	0.226

We conducted additional sensitivity MR analyses using alternative genetic instruments considering results from two recent GWAS on 25(OH)D [23, 24]. Both of these additional MR analyses confirmed the results from our main analysis since the overall estimates, calculated by IVW or MR-Egger, did not reveal an overall association of genetically predicted 25(OH)D levels on the risk of ADHD (Tables S1 and S2, Figures S4 and S5). Weighted Median, Simple Mode, and Weighted Mode confirmed the lack of associations (Table S1 and S2).

## MR assessing ADHD effects on 25(OH)D

To test reverse causality, we performed a MR analysis where we considered the risk of ADHD as exposure and 25(OH)D as the outcome. Data on the association of the 12 selected SNPs with ADHD and with 25(OH)D is presented in Table 3. We used effect estimates and P-values that were calculated for Europeans. For seven SNPs, which were not included in GWAS for 25(OH)D we used proxy-SNPs. For SNPs rs11420276 and rs5886709 no proxies where available. Since we had to exclude four palindromic SNPs (Table 3: palindromic + ambiguous = true), we were able to include six SNPs in this MR analysis. With *F* = 21,867.14, the F-statistic indicates strong instrumental variables (for F-statistic calculation, the proportion of variance (*R*^2^ = 0.216) was considered as determined using those cohorts of European ancestry and all 12 genome-wide significant SNPs in the ADHD GWAS). There was no evidence for pleiotropy (MR-Egger intercept: 0.0043, SE = 0.0044; *p* = 0.379). Heterogeneity tests were not significant (Cochran's Q (*df* = 4) = 4.473, *p* = 0.346) and MR-PRESSO also revealed no significant outliers.

**Table 3:** SNPs associated with ADHD (European ancestry) and natural-log transformed 25(OH)D levels

SNP	Chr	Position	ADHD						25(OH)D					Palindromic + ambigous
			A1	A2	A1 freq	OR	SE	*P* value	A1	A2	Beta	SE	*P* value	
*rs11420276*^*a*^	*1*	*44184192*	*G*	*GT*	*0.696*	*1.11305*	*0.0149*	*6.452e*−*13*						
rs11591402	10	106747354	A	T	0.224	0.91174	0.0164	1.76e−08	A	T	0.0012	0.0025	0.6195	True
*rs1222063*^*b*^	*1*	*96602440*	*A*	*G*	*0.328*	*1.10098*	*0.0174*	*3.068e*−*08*						
^b^rs1222067	1	96597502	A	C	0.688	0.92635	0.0155	7.908e−07	A	C	0.0008	0.0026	0.7464	False
rs1427829	12	89760744	A	G	0.434	1.08567	0.0136	1.349e−09	A	G	0.0027	0.0021	0.1974	False
*rs212178*^*c*^	*16*	*72578131*	*A*	*G*	*0.883*	*0.88950*	*0.0205*	*1.198e*−*08*						
^c^rs12596294	16	72587093	A	T	0.098	1.12008	0.0207	4.157e−08	A	T	0.0029	0.0034	0.3873	True
rs281324	15	47754018	T	C	0.531	0.92450	0.0135	6.684e−09	T	C	0.0024	0.0021	0.2426	False
*rs28411770*^*d*^	*4*	*31151456*	*T*	*C*	*0.651*	*1.08992*	*0.0151*	*1.152e*−*08*						
^d^rs7674790	4	31149277	A	T	0.610	1.07455	0.014	2.996e−07	A	T	0.0002	0.0022	0.9348	True
*rs4858241*^*e*^	*3*	*20669071*	*T*	*G*	*0.622*	*1.08567*	*0.0143*	*8.172e*−*09*						
^e^rs17808771	3	20691823	A	T	0.758	1.07326	0.0153	3.773e−06	A	T	0.0008	0.0024	0.7341	True
rs4916723	5	87854395	A	C	0.573	0.92515	0.0138	1.807e−08	A	C	− 0.0016	0.0021	0.4515	False
*rs5886709*^*f*^	*7*	*114086133*	*G*	*GTC*	*0.463*	*1.07993*	*0.0137*	*2.056e*−*08*						
*rs74760947*^*g*^	*8*	*34352610*	*A*	*G*	*0.957*	*0.83560*	*0.0317*	*1.393e*−*08*						
^g^rs6990255	8	34126948	T	C	0.0586481	1.18270	0.0315	1.03e−07	T	C	− 0.0058	0.0043	0.1779	False
rs9677504	2	215181889	A	G	0.109	1.12019	0.0213	9.829e−08	A	G	0.0012	0.0033	0.7171	False

*Chr* chromosome, *A1* allele 1, *A2* allele 2, *freq* allele frequency, *OR* odds ratio, *SE* standard error; *palindromic* + *ambiguous true* this SNPs are palindromic and ambiguous (excluded after harmonization)

^a^No Proxy-SNP available for rs11420276

^b^For rs1222063: proxy rs1222067; distance: − 4938; *R*^2^:0.679350

^c^For rs212178: proxy rs12596294; distance: 8962; *R*^2^: 0.924371

^d^For rs28411770: proxy rs7674790; distance: − 2179; *R*^2^: 0.797643

^e^rs4858241: proxy rs17808771; distance:22,752; *R*^2^: 0.646552

^f^No proxy-SNP available for rs5886709

^g^For rs74760947: proxy rs6990255; distance: − 225,662; *R*^2^: 0.947204

**Fig. 3:**
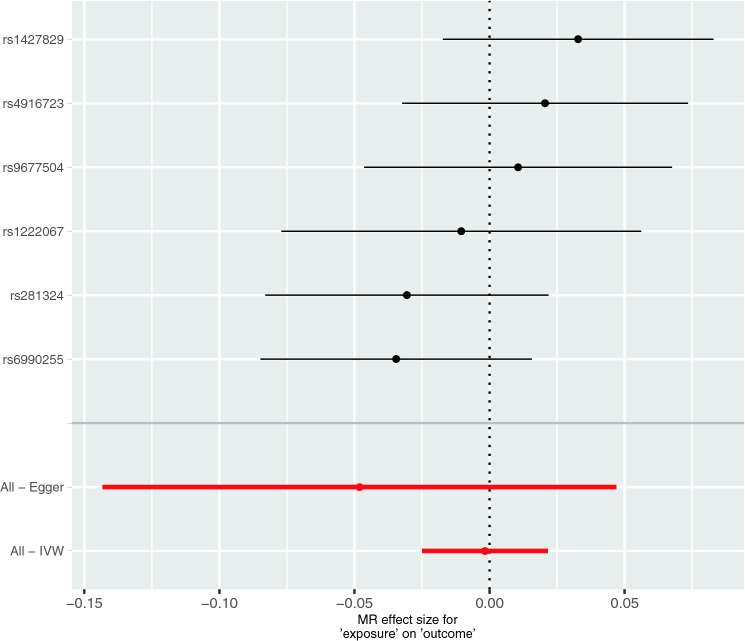
Results of the single and multi-SNP analyses for the SNP effect of risk for ADHD on natural-log transformed 25(OH)D levels. The black lines visualize the results of single SNP analyses; the red lines visualize the results of the multi-SNP analysis

No single SNP showed a causal effect of ADHD on 25(OH)D. Further, the overall effect was not significant (IVW method: *β* = − 0.002, *p* = 0.887; MR Egger method: *β* = − 0.048, *p* = 0.377) (Table 4, Figs. 3, S6 and S7). Sensitivity analyses using Weighted Median, Simple Mode, and Weighted Mode (Table 4, Figures S6 and S7) and MR-PRESSO (*β* = − 0.001, SD = 0.019, *p* = 0.892) also revealed no significant overall effect. Leave-one-out analysis also showed no effect (Figure S8). Additionally, we performed a sensitivity analysis with proxy SNPs, which were neither palindromic nor ambiguous (Table S3). This analysis also showed no causal effect of ADHD on 25(OH)D (Table S4, Figure S9).

**Table 4:** Results of single SNP MR analyses and the overall causal effect of ADHD on 25(OH)D levels using different statistical methods

SNP	Beta	SE	*P* value
rs1222067	− 0.010	0.034	0.758
rs1427829	0.033	0.026	0.199
rs281324	− 0.031	0.027	0.253
rs4916723	0.0206	0.027	0.446
rs6990255	− 0.035	0.026	0.177
rs9677504	0.011	0.029	0.716
Inverse variance weighted	− 0.002	0.012	0.887
MR Egger	− 0.048	0.049	0.377
Weighted median	0.002	0.015	0.900
Simple mode	0.015	0.026	0.571
Weighted mode	0.019	0.027	0.514

## Discussion

The main finding of this MR study was that there was no genetic evidence for a causal effect of 25(OH)D levels on the risk of ADHD. Additionally, our analysis did also not indicate a causal relationship in the opposite direction, i.e. ADHD did not have an effect on 25(OH)D levels.

Interestingly, one of the five SNPs used for the definition of the IV (rs12785878) even indicated a significant positive effect of 25(OH)D on the risk of ADHD, i.e. higher risk for ADHD at higher 25(OH)D levels (*p* = 0.024). Also, one of the leave-one-out sensitivity analyses indicated a significant overall effect in the same direction, when rs3755967, indicating an inverse association, was excluded from the analysis. However, it has to be kept in mind that rs12785878 would not survive correction for multiple testing (five SNPs). Additionally, underlying pathophysiologic mechanisms of a harmful effect of 25(OH)D on ADHD are difficult to understand. Thus, this surprising finding underlines the fact that additional GWAS on 25(OH)D and ADHD are still urgently needed to increase the number of genome-wide significant SNPs and to further improve the genetic instrument for future MR studies.

During infancy, recommendations in Germany include routine vitamin D supplementation [36]. For children aged > 2 years, the German Society for Pediatrics and Adolescent Medicine recently concluded that such routine supplementation is not recommended for children who do not have risk factors and chronic diseases which are associated with calcium or vitamin D resorption disorders [3]. Findings from the meta-analyses of observational studies on ADHD might raise concern regarding this recommendation since 25(OH)D levels were found to be lower in ADHD patients compared to healthy controls [37, 38]. Additionally, low 25(OH)D cord blood levels were prospectively associated with higher ADHD scores in toddlers [39] indicating a potential role of vitamin D supply in the perinatal period and, thus, perhaps also for long-term prevention of this neuro-developmental disorder in later life. In general, RCTs are regarded as the gold standard to prove causality, but the few RCTs on vitamin D and ADHD were restricted to the therapeutic effects of an already manifest disorder. Considering results from four RCTs, a recent meta-analysis suggested that vitamin D supplementation could be one treatment option, but all included RCTs had some considerable limitations such as small sample size (35–96 subjects) and a low to very-low quality of evidence according to GRADE criteria [10]. Accordingly, the authors requested well-designed RCTs to confirm the efficacy of vitamin D supplementation for children and adults with ADHD [10].

Overall, current evidence seems to be in line with findings on 25(OH)D and depression: Here, observational studies also showed positive associations [40], while the potential effects of vitamin D supplementation might be restricted to subjects with clinically significant depressive symptoms [41]. While preventive effects on depression onset were only assessed in a few RCTs with follow-up periods of more than one year, MR studies on depression did not support the suggested preventive role of 25(OH)D [16, 17]. Now, results from our current MR study did also not confirm a causal effect of 25(OH)D on ADHD. In general, it has been argued that conflicting results between observational studies and MR studies might indicate bias from residual confounding in observational studies or reverse causation [12]. The latter would imply that suffering from the disease would have negative effects on 25(OH)D, e.g. due to social withdrawal and reduced sunlight exposure. While such reverse effects on 25(OH)D are plausible in internalizing disorders such as depression, it seems less obvious in the case of ADHD. Also, results from our reverse MR analysis did not reveal any evidence for reverse causality. Nevertheless, residual confounding in observational studies still seems reasonable since low 25(OH)D could be a marker of ill health in general [42].

To the best of our knowledge, our study is the first two-sample MR study on causal relationships between 25(OH)D levels and ADHD. A strength of our study is the consideration of recent, large-scaled GWAS on both vitamin D metabolism (*n* = 79,366) [19] and on ADHD (*n* = 20,183 cases) [20]. Accordingly, our study had sufficient statistical power which allowed the detection of OR > 1.27 per 1 standard deviation decrease in natural-log transformed 25(OH)D levels with a statistical power of 100% (OR > 1.159 with 80% power). However, we cannot rule out small preventive effects below this threshold. Despite using the latest available GWAS with the largest sample sizes and highest number of identified SNPs for our data analyses, it must be kept in mind that this field of genetic research has a substantial turnover and findings from our MR study should not be interpreted as final results. Actually, while this paper was under review, two GWAS on 25(OH)D concentrations, each with more than 400,000 European study participants, were published. These GWAS revealed 143 and 138 genome-wide significant SNPs, respectively [23, 24]. Sensitivity MR analyses considering these findings for the definition of the IV also showed no significant association between genetically predicted 25(OH)D concentrations and ADHD. Accordingly, our result that there is no evidence for a causal effect of 25(OH)D on ADHD appears robust.

A limitation of our MR study is that only six SNPs, which explain 2.8% of the variance of 25(OH)D levels, could be considered for the definition of the IV. One of these SNPs was ambiguous and palindromic and had thus had to be excluded. Thus, our instrument might be regarded as weak and bias the association with ADHD. However, the F-statistics indicated that our genetic instrument was sufficiently strong. Furthermore, our sensitivity analyses using a higher number of SNPs as genetic instrument confirmed results from our main analysis.

Another general issue of MR studies is horizontal pleiotropy, i.e. an association between the MR instrument and the outcome of interest via pathways other than the suggested exposure [12]. Even though this issue cannot be completely ruled out in MR studies, the MR Egger intercept and MR-PRESSO analyses revealed no indication of pleiotropy.

When interpreting the results, it is important to note that the vitamin D GWAS did not focus on individuals with low (or high) levels of 25(OH)D. Since our MR analysis assumed a linear relationship between exposure and outcome, the results might not apply to people with a severe 25(OH)D deficiency, but to the general population [11]. Additionally, it must be kept in mind that the instrumental variable was not derived from GWAS focussing on childhood 25(OH)D. In conclusion, our MR study using data from large-scaled genetic studies provides initial evidence that 25(OH)D might not have substantial preventive effects on ADHD. Well-designed, large-scaled RCTs would be important to definitely evaluate the preventive capacity of routine supplementation during infancy. Complex requirements regarding study design such as long intervention and follow-up periods, especially in diseases with low incidence [43], might be one explanation why such RCTs are still lacking.

## Electronic supplementary material

Below is the link to the electronic supplementary material.

Supplementary file1 (DOCX 10573 KB)

Supplementary file2 (DOCX 33 KB)
