# Supplementary material for: A mendelian randomization study on causal effects of 25(OH)vitamin D levels on attention deficit/hyperactivity disorder
Source: Eur J Nutr. 2020 Nov 27;60(5):2581–91. doi: 10.1007/s00394-020-02439-2 (PMC8275531; doi:10.1007/s00394-020-02439-2)
Supplement: Supplementary file 1 — Supplementary file1 (DOCX 10573 KB) [file 394_2020_2439_MOESM1_ESM.docx]

**Supplementary Figures**


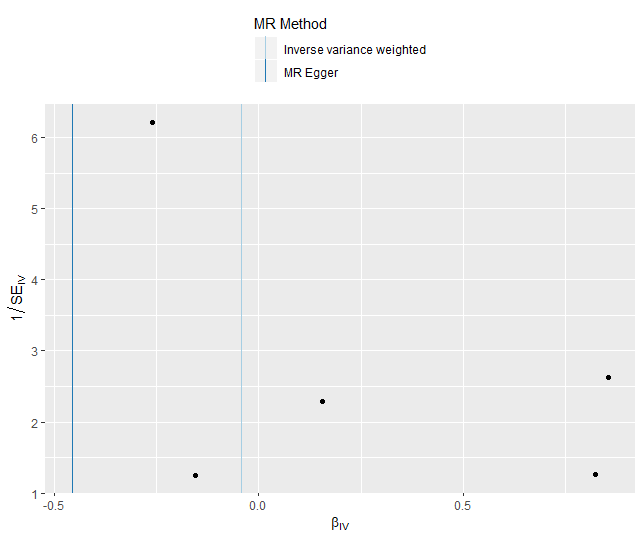


**Figure S1.** Funnel plot for the MR analysis with level of 25(OH)D as exposure and risk for ADHD as outcome

**
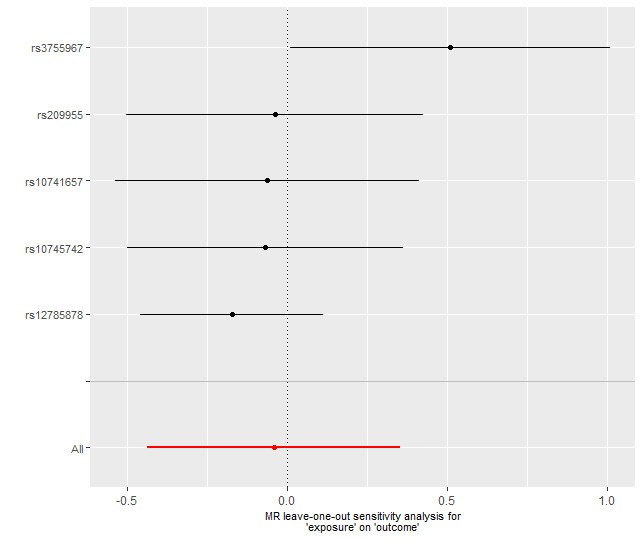
**

**Figure S2.** Leave-one-out approach for the MR analysis with level of 25(OH)D as exposure and risk for ADHD as outcome.


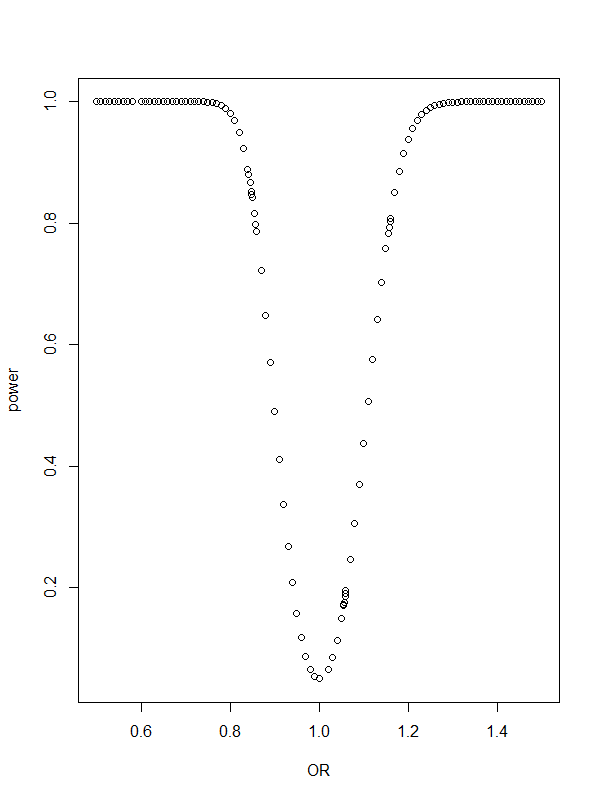


**Figure S3.** Calculated power to detect a true causal effect of a relative difference between 1% and 20% per 1 standard deviation in 25(OH)D levels. Power 0.80 for OR=1.159; The following data were used for the calculation: K=0.358 (ADHD), N=53293 (ADHD), R^2^xy=0.0284 (25(OH)D)


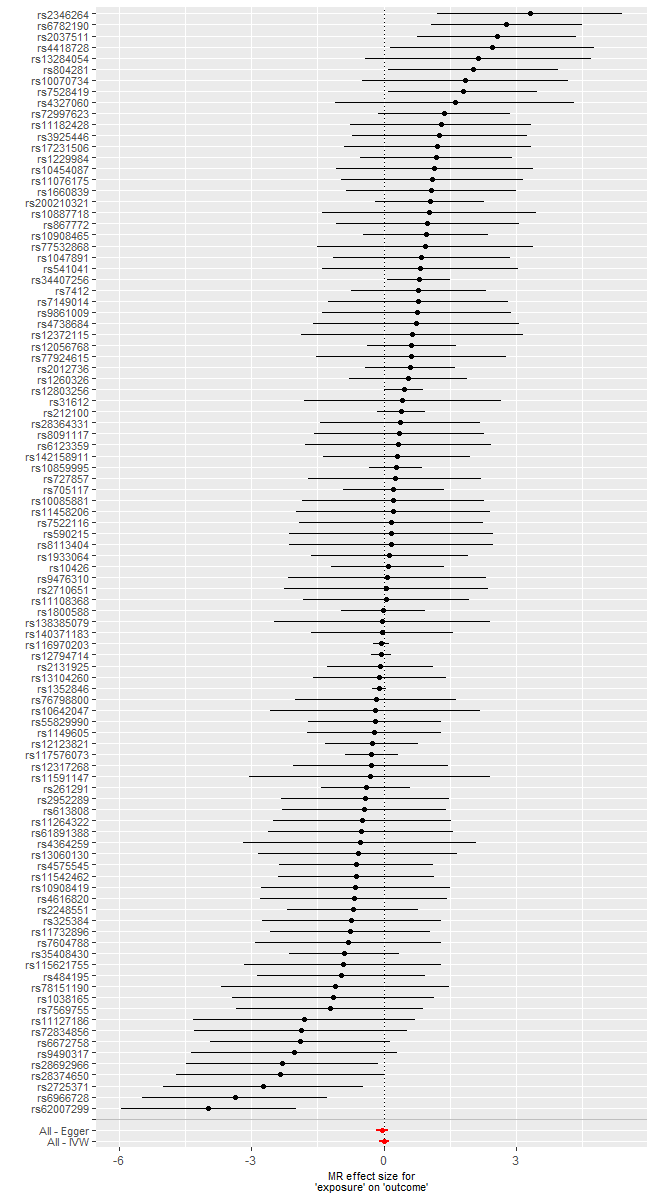


**Figure S4.** Results of the single and multi-SNP analyses using 100 from 143 SNPs identified genome-wide significant hits from Revez et al. 2020 as genetic instrument


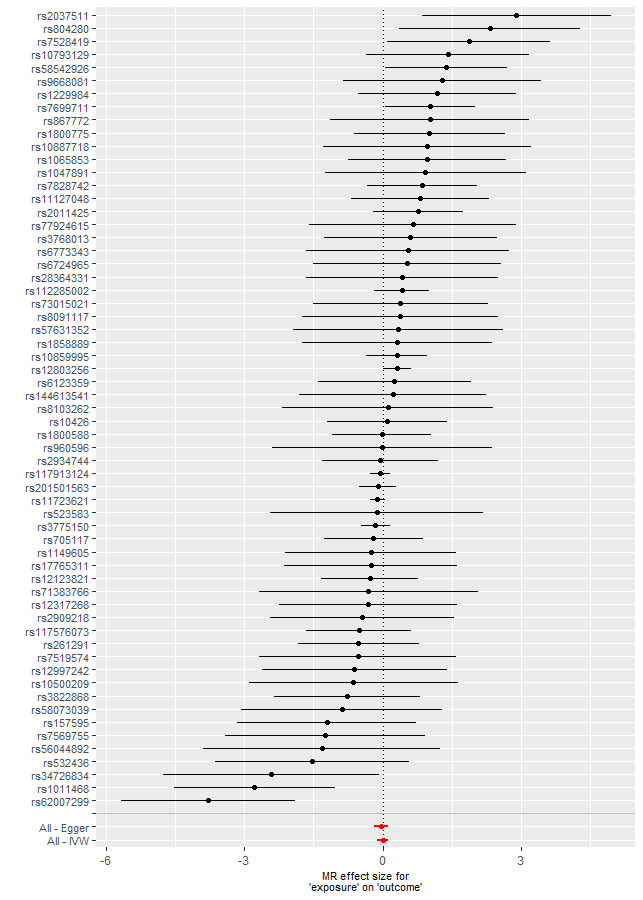


**Figure S5.** Results of the single and multi-SNP analyses using 61 from 138 SNPs identified genome-wide significant hits from Manousaki et al. 2020 as genetic instrument

**
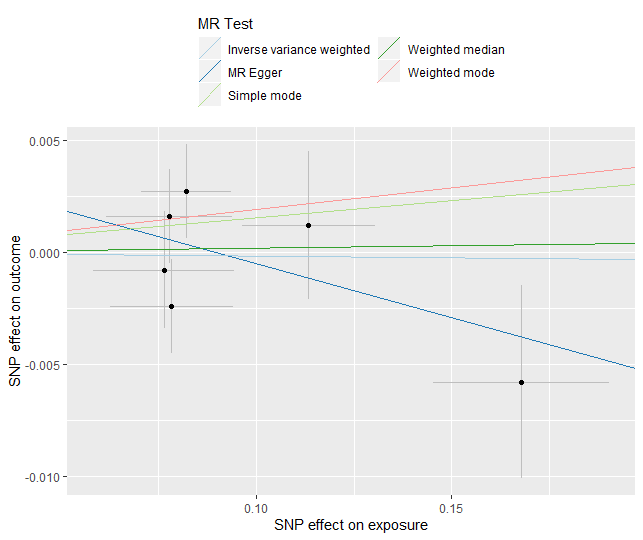
**

**Figure S6** Scatter plots of genetic associations with ADHD against natural-log transformed 25(OH)D levels using different MR methods. The slopes of each line represent the causal association for each method.

**
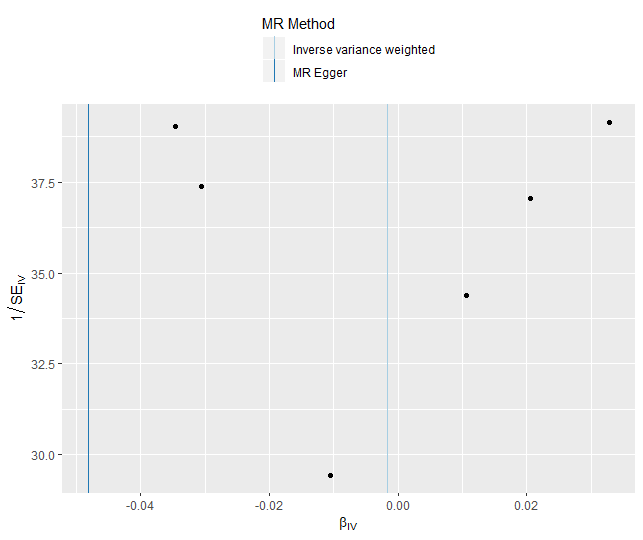
**

**Figure S7.** Funnel plot for the MR with risk for ADHD as exposure and level of 25(OH)D as outcome

**
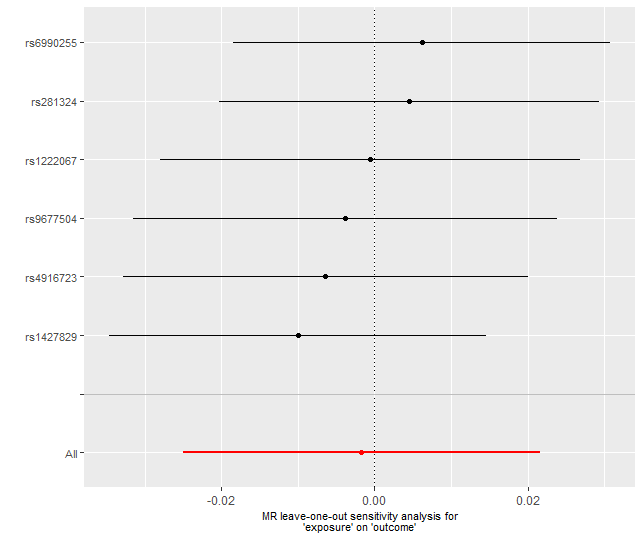
**

**Figure S8.** Leave-one-out approach for the MR analysis with risk for ADHD as exposure and level of 25(OH)D as outcome.


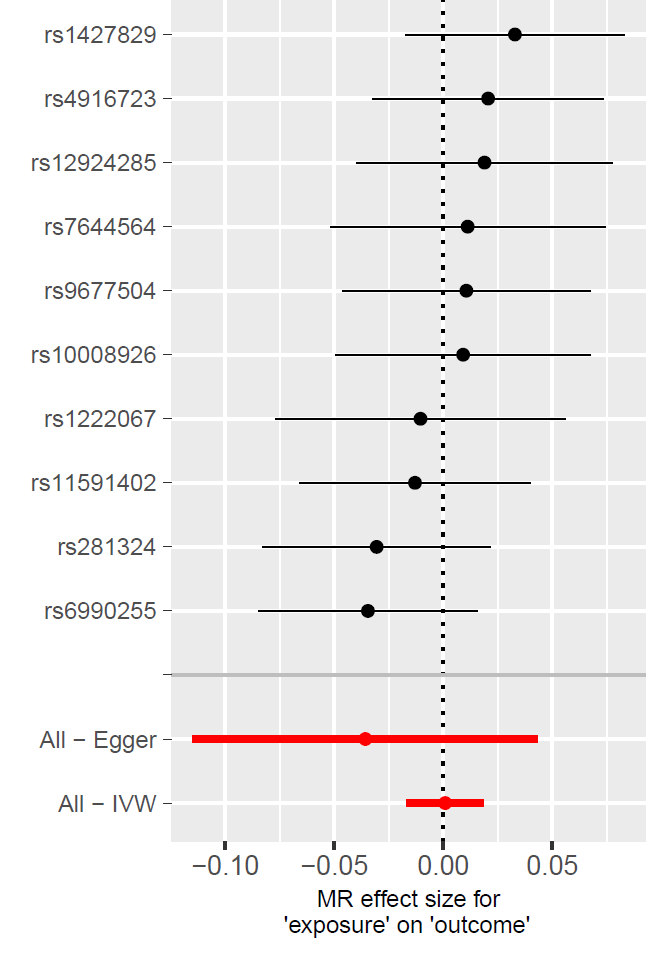


**Figure S9.** Results of the single and multi-SNP analyses for the SNP effect of risk for ADHD on natural-log transformed 25(OH)D levels. SNPs, which had no results for 25(OH)D, were replaced with proxy SNPs. The best non-palindromic and non-ambiguous SNPs were selected. The black lines visualize the results of single SNP analyses; the red lines visualize the results of the multi SNP analysis.
