# Supplementary material for: A mendelian randomization study on causal effects of 25(OH)vitamin D levels on attention deficit/hyperactivity disorder
Source: Eur J Nutr. 2020 Nov 27;60(5):2581–91. doi: 10.1007/s00394-020-02439-2 (PMC8275531; doi:10.1007/s00394-020-02439-2)
Supplement: Supplementary file 2 — Supplementary file2 (DOCX 33 KB) [file 394_2020_2439_MOESM2_ESM.docx]

| **Table S1.** Results of MR analysis on the overall causal effect of 25(OH)D levels on ADHD using 100 from 143 SNPs identified genome-wide significant hits from Revez et al. 2020 as genetic instrument* | | | |
| --- | --- | --- | --- |
| **Method** | **Beta** | **SE** | **P-Value** |
| MR Egger | -0.029 | 0.069 | 0.673 |
| Weighted median | -0.062 | 0.063 | 0.330 |
| Inverse variance weighted | 0.009 | 0.055 | 0.864 |
| Simple mode | 0.049 | 0.202 | 0.810 |
| Weighted mode | -0.062 | 0.057 | 0.277 |
| *only those SNPs were considered which were both available in the ADHD GWAS and not ambiguous or palindromic  There was no evidence for pleiotropy considering MR-Egger intercept (0.0024, SE=0.0026; p=0.362), but for heterogeneity (Cochran's Q (df=98)=151.5, p=0.00042). The global test of MR PRESSO was significant (p=0.0003), outlier suspicion: rs62007299), outlier-corrected beta=0.0169, SD=0.052, p=0.746. | | | |

| **Table S2.** Results of MR analysis on the overall causal effect of 25(OH)D levels on ADHD using 61 from 138 SNPs identified genome-wide significant hits from Manousaki et al. 2020 as genetic instrument* | | | |
| --- | --- | --- | --- |
| **Method** | **Beta** | **SE** | **P-value** |
| MR Egger | -0.033 | 0.079 | 0.681 |
| Weighted median | -0.106 | 0.069 | 0.122 |
| Inverse variance weighted | 0.007 | 0.061 | 0.915 |
| Simple mode | 0.039 | 0.185 | 0.833 |
| Weighted mode | -0.062 | 0.059 | 0.298 |
| *only those SNPs were considered which were both available in the ADHD GWAS and not ambiguous or palindromic | | | |
| There was no evidence for pleiotropy considering MR-Egger intercept (0.0027, SE=0.0034; p=0.432), but for heterogeneity (Cochran's Q (df=59)= 92.81, p=0.0033). The global test of MR PRESSO was significant (p=0.0033), outlier suspicion: rs3768013), outlier-corrected beta=0.016, SD=0.056, p=0.773. | | | |

| **Table S3.** Genome-wide significant SNPs for ADHD (exposure) and their associations with natural log-transformed 25(OH)D levels (outcome). SNPs, which had no results for 25(OH)D, were replaced with proxy SNPs. The best non-palindromic and non-ambiguous SNPs were selected. | | | | | | | | | | | | |
| --- | --- | --- | --- | --- | --- | --- | --- | --- | --- | --- | --- | --- |
|  |  | ADHD | | | | | | 25(OH)D | | | | |
| SNP* | SNP in MR | A1 | A2 | A1 Freq | OR | SE | P-value | A1 | A2 | Beta | SE | P-value |
| rs11591402 | rs11591402 | A | T | 0.224 | 0.91174 | 0.0164 | 1.76e-08 | A | T | 0.0012 | 0.0025 | 0.6195 |
| rs1427829 | rs1427829 | A | G | 0.434 | 1.08567 | 0.0136 | 1.349e-09 | A | G | 0.0027 | 0.0021 | 0.1974 |
| rs4916723 | rs4916723 | A | C | 0.573 | 0.92515 | 0.0138 | 1.807e-08 | A | C | -0.0016 | 0.0021 | 0.4515 |
| rs9677504 | rs9677504 | A | G | 0.109 | 1.12019 | 0.0213 | 9.829e-08 | A | G | 0.0012 | 0.0033 | 0.7171 |
| rs281324 | rs281324 | T | C | 0.531 | 0.92450 | 0.0135 | 6.684e-09 | T | C | 0.0024 | 0.0021 | 0.2426 |
| rs1222063 | rs1222067 | A | C | 0.688 | 0.92635 | 0.0155 | 7.908e-07 | A | C | 0.0008 | 0.0026 | 0.7464 |
| rs212178 | rs12924285 | A | G | 0.094 | 1.12345 | 0.021 | 3.185e-08 | A | G | 0.0022 | 0.0035 | 0.5204 |
| rs4858241 | rs7644564 | A | G | 0.759 | 1.07412 | 0.0152 | 2.787e-06 | A | G | 0.0008 | 0.0023 | 0.7117 |
| rs74760947 | rs6990255 | T | C | 0.059 | 1.18270 | 0.0315 | 1.03e-07 | T | C | -0.0058 | 0.0043 | 0.1779 |
| rs28411770 | rs10008926 | A | G | 0.32 | 0.92598 | 0.0151 | 3.353e-07 | A | G | -0.0007 | 0.0023 | 0.7646 |

* genome-wide significant SNP for ADHD

**Table S4.** Results of single SNP MR analyses and the overall causal effect of risk of ADHD on natural-log transformed 25(OH)D levels. SNPs, which had no results for 25(OH)D, were replaced with proxy SNPs. The best non-palindromic and non-ambiguous SNPs were selected.

|  | | | |
| --- | --- | --- | --- |
| SNP | Beta | SE | P-value |
| rs10008926 | 0.009 | 0.030 | 0.761 |
| rs11591402 | -0.013 | 0.027 | 0.631 |
| rs1222067 | -0.011 | 0.034 | 0.758 |
| rs12924285 | 0.019 | 0.030 | 0.530 |
| rs1427829 | 0.033 | 0.026 | 0.199 |
| rs281324 | -0.031 | 0.027 | 0.253 |
| rs4916723 | 0.021 | 0.027 | 0.446 |
| rs6990255 | -0.035 | 0.026 | 0.177 |
| rs7644564 | 0.011 | 0.032 | 0.728 |
| rs9677504 | 0.011 | 0.030 | 0.716 |
| Inverse variance weighted | 0.001 | 0.009 | 0.923 |
| MR Egger | -0.036 | 0.040 | 0.402 |
| Weighted median | 0.010 | 0.012 | 0.396 |
| Simple mode | 0.014 | 0.021 | 0.534 |
| Weighted mode | 0.015 | 0.021 | 0.507 |
